# Case report: Idiopathic pulmonary fibrosis induced by nab-paclitaxel: A rare complication

**DOI:** 10.3389/fphar.2023.1094844

**Published:** 2023-02-23

**Authors:** Jiahui Shen, Zhongyong Wen, Jingxia Lin, Huiwen Su

**Affiliations:** Gynaecology of The Sixth Affiliated Hospital, School of Medicine, South China University of Technology, Foshan, China

**Keywords:** ovarian cancer, nab-paclitaxel, idiopathic pulmonary fibrosis, IPF, TRAE

## Abstract

**Background:** Ovarian cancer is one of the deadliest gynecological cancers, with the most advanced disease and poor survival. Although BRCA genes play a key role in maintaining genomic stability and providing the possibility of clinically individualized treatments, with the emergence of new and more appropriate treatment options, new treatment–related adverse events are challenging and difficult for clinicians.

**Case presentation:** An 80-year-old Chinese woman was diagnosed with stage IIIC ovarian high-grade serous adenocarcinoma (CT3cN1MX) with BRCA2 as the causative gene. She underwent three courses of neoadjuvant chemotherapy with nab-paclitaxel 400 mg and carboplatin 450 mg before surgery. Chest HRCT prior to chemotherapy demonstrated bilateral interstitial pneumonia. During chemotherapy, there were four episodes of dry cough, shortness of breath, dyspnea, and three episodes of bone marrow suppression. The symptoms became intermittent and progressively worse, and after three sessions of empirical cough and phlegm relief, oxygen inhalation, corticosteroids, anti-infectives, and leukopenia therapy, the symptoms became intermittent and progressively worse. The diagnosis of idiopathic pulmonary fibrosis came a week after the third round of chemotherapy. After a strong dose of corticosteroids and nintedanib anti-fibrosis therapy, the pulmonary symptoms abated, and intermediate tumor starvation was performed. The combination therapy was subsequently discontinued, and the patient experienced significant relief from pulmonary symptoms. Treatment response was positive following single-agent nab-paclitaxel 400 mg chemotherapy in combination with nintedanib 150 mg anti-fibrosis therapy.

**Conclusion:** In this report, we describe a rare case of idiopathic pulmonary fibrosis associated with the use of nab-paclitaxel and carboplatin in ovarian cancer. During treatment, it is necessary to maintain a high level of vigilance for patients with interstitial pneumonia and engage the attention of clinicians to improve medication safety. Early diagnosis and anti-fibrosis therapy can reverse lung damage.

## Introduction

Ovarian cancer (OC) is one of the three predominant malignant tumors of the female reproductive system, is difficult to diagnose at its onset, and lacks practical screening tests. Ovarian cancer, the fifth leading cause of cancer death in women, has a shallow cure rate. Seventy percent of the patients are diagnosed with advanced disease (Reid et al., 2021; Shaik et al., 2021). Recent studies have shown that maintaining genomic stability depends on the BRCA gene. In addition, the emergence of new drugs, such as bevacizumab and poly(adenosine diphosphate-ribose) polymerase (PARP) inhibitors, provides an opportunity and the best solution for the stratified treatment of OC patients (Arora et al., 2021). However, these regimens are associated with higher rates of original treatment–related adverse events (TRAEs) in OC patients. Therefore, carboplatin (CBP) combined with nab-paclitaxel (nab-PTX) chemotherapy still plays a crucial role in the first-line treatment of OC patients (Armstrong et al., 2021).

Nab-PTX is a novel, solvent-free formulation of the protein-stabilized paclitaxel. It adsorbs to tumor cells through a specific protein (SPARC), enters the cell, and releases the cytotoxic drug, effectively increasing the dose of paclitaxel. There is no need for pretreatment to prevent allergy before medication, which shortens the medication time (Kundranda and Niu, 2015). It was approved in 2014 as the first-line treatment for various locally advanced or metastatic malignancies and has shown positive results in ovarian cancer patients (Yang and Fu, 2022). The adverse reactions of Nab-PTX chemotherapy mainly involve the hemolymph system, nervous system, musculoskeletal system and gastrointestinal system (Otsubo et al., 2017). Idiopathic pulmonary fibrosis (IPF) is rare in lung cancer (LC) chemotherapy patients (Fukuizumi et al., 2019), but not in OC chemotherapy patients.

The application and continuous improvement of anti-tumor drugs also mean that new TRAEs, such as niraparib, can cause serious complications such as pulmonary embolism (Wei et al., 2022). Although nab-PTX, an improved new paclitaxel drug, rarely causes IFP, it increases the associated risks and is even life-threatening. During clinical application, we should be highly alert to nab-PTX use in advanced cancer patients with interstitial pneumonia (IIP). To maximize efficacy and reduce adverse events, appropriate formulation and use of the drug remain major clinical tasks.

## Case description

**FIGURE 1:**
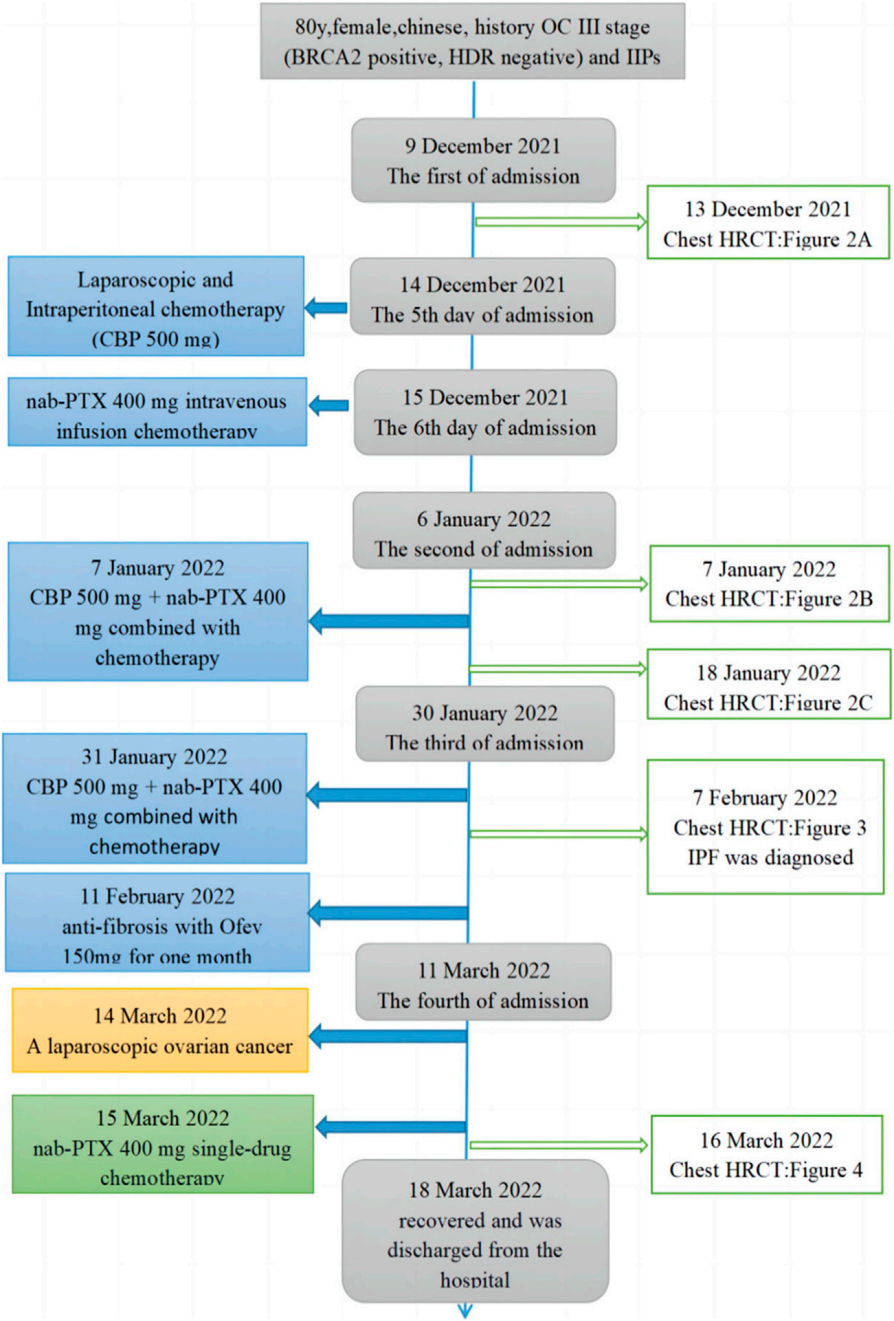
Timeline and treatment process. The blue line in the figure indicates time points in the progression of the patient’s condition. The boxes on the left indicate treatment events. The connect boxes indicates HRCT examinations of the chest.

**FIGURE 2:**
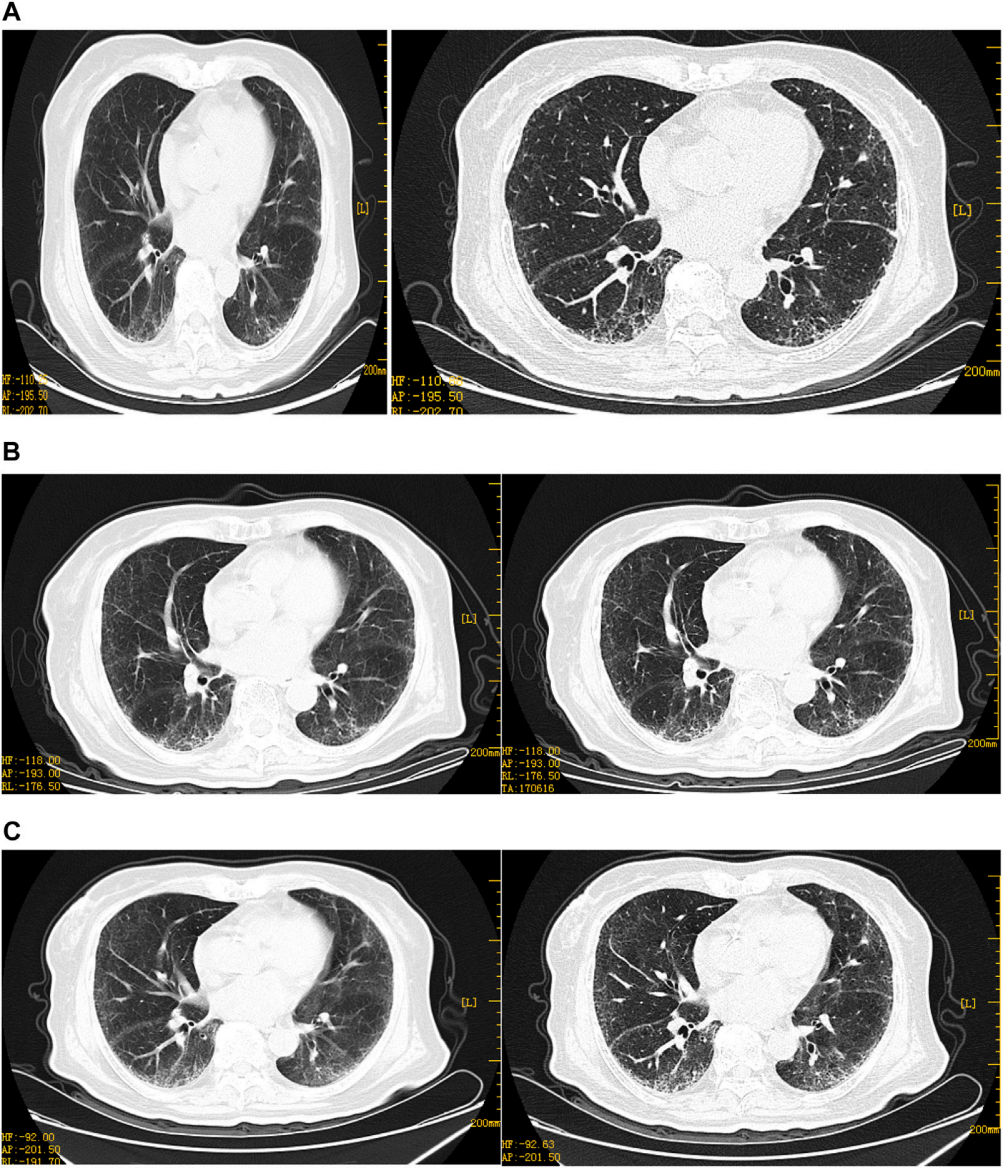
Representative HRCT images of the lungs before neoadjuvant chemotherapy. **(A)**HRCT on 13 December 2021: multiple grid-like density increases in and around the subpleural and lung bases, blurred borders, peripheral distribution, double lower lung, subpleural and lung base and peripheral areas, trachea, and lobes, bronchial opening segments displayed, no stenosis, no significant expansion in the distal part, no fibrosis. **(B)**HRCT on 7 January 2022, multiple grid-like density increases under the pleura of both the lungs, slightly more than before, the boundaries blurred, and multiple hair-like and small patchy blurred shadows are visible in both lungs. On the 52nd day, due to inflammation of the lungs, **(C)**HRCT on 18 January 2022, multiple grid-like density increases under the pleura of both the lungs increased when compared with the second time, peripheral distribution, considering the possibility of interstitial inflammation of both the lungs, aggravation of lung symptoms during chemotherapy. Diagnosis of IPF by respiratory study therapy.

**FIGURE 3:**
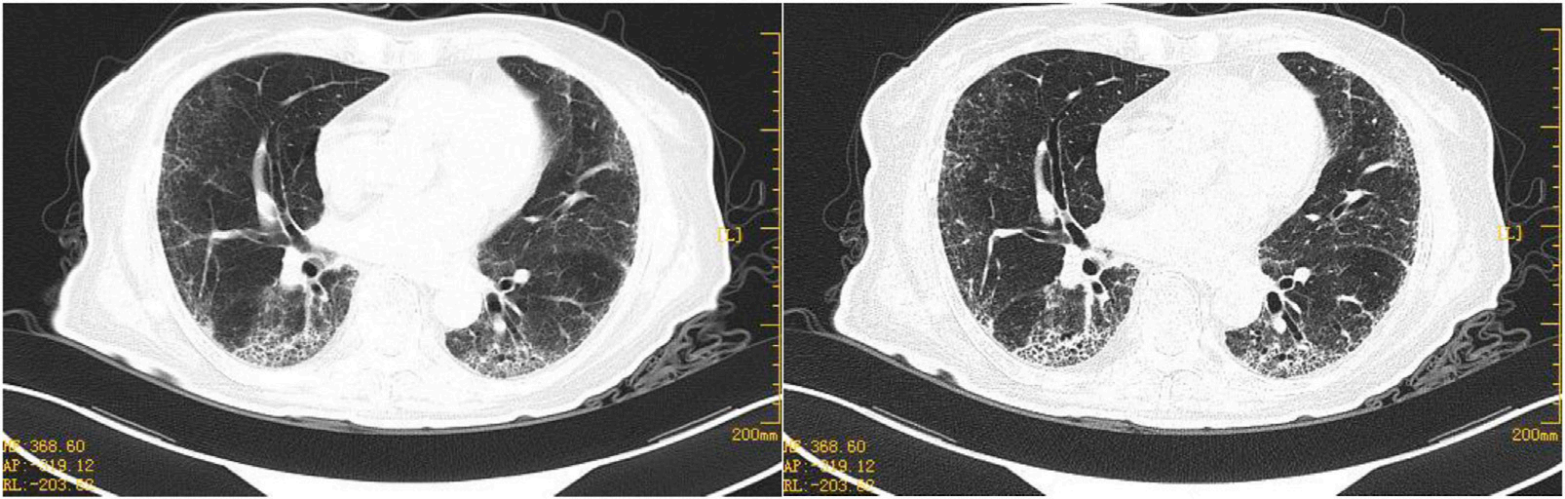
Representative HRCT images after 1 week of anti-fibrosis treatment. HRCT on 7 February 2022, bilateral pulmonary interstitial pneumonia and pulmonary fibrosis, both the lungs are primarily scattered in spot-like, cord-like dense opacities, with multiple grid-like density increases seen below the pleura of both lungs and blurred boundaries.

**FIGURE 4:**
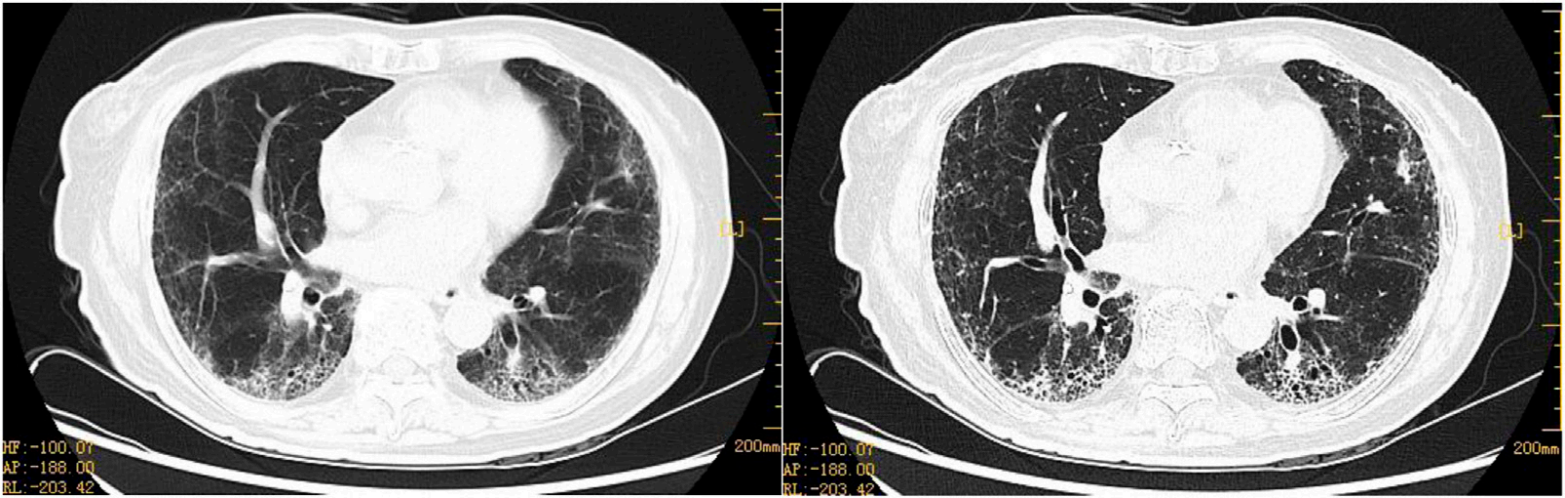
Representative HRCT images of ovarian cancer after ablation surgery with nab-PTX 400 mg chemotherapy. Postoperative pulmonary symptoms not considered to be an adverse reaction caused by nab-PTX, which was administered with 400 mg of chemotherapy on the first postoperative day following OC reduction. HRCT on 16 March 2022, 36 h after chemotherapy: chronic bronchitis with emphysema, interstitial lung lesions, and bronchiectasis in both lower lobes. Both lobes have chronic bronchitis, emphysema, interstitial lung lesions, and bronchiectasis.

An 80-year-old female was diagnosed with OC III (BRCA2 positive and HDR negative) with interstitial lung disease (ILD) but without pulmonary symptoms. She had no history of smoking or drinking, exposure to dust, radiation, and poisons, distinct personal history, family history of tumors, or genetic history. The course of its treatment is shown in Figure 1. At first, the patient presented with abdominal dimensions, pelvic tumors, and malignant ascites. Abdominal MRI showed ascites and a round-like mixed signal shadow in the right right accessory area, measuring about 41 × 29 × 23 mm, considering the possible origin of ovary. Chest HRCT showed multiple grid-like over-dense shadows under the pleura of both lungs on the fourth day of admission. The boundary was fuzzy, presenting interstitial inflammation of both the lungs (Figure 2A), but the patient had no pulmonary symptoms. Due to rapid disease progression on the sixth day of admission, it was decided to communicate with the anesthesiologist and perform laparoscopic exploration and pelvic mass biopsy under general anesthesia. Biopsy confirmed the diagnosis of high-grade serous adenocarcinoma with malignant tumor cells seen in the ascites—operational pathology stage: IIIC stage, where surgery is difficult to reach R0. After discussions with the family, it was decided to perform an intermediate tumor reduction procedure. CBP 500 mg intra-peritoneal chemotherapy was administered during surgery, and nab-PTX 400 mg intravenous chemotherapy was issued the first day after surgery. The process went smoothly, with mild side effects. In the first week after chemo, mild cough, expectorant, minor, white, and type II bone marrow suppression began to appear. After a week of treatment for leukocytosis, cough relief, and phlegm resolution, the symptoms improved. In the third week, the patient continued to have a mild but non-obvious cough, and the second course of chemotherapy was continued as scheduled. Before chemotherapy, chest HRCT showed multiple grid-like shadows with increased density below the pleura in both the lungs. Numerous cord-like clouds were seen, which were more evident than before, mainly in the lower lungs, and interstitial pneumonia was considered (Figure 2B). Furthermore, CBP 500 mg + nab-PTX 400 mg were continued, combined with the second chemotherapy. Coughing and expectoration returned a week after chemotherapy. Symptoms are slightly worse than they were before, with a small amount of white, sticky phlegm and a challenging cough. They improved slightly after the treatment with cough drops and expectorants. During the second week after chemotherapy, symptoms worsened, primarily due to dry cough, chest tightness, shortness of breath, increased post-exercise activity, and grade III bone marrow suppression. A repeat HRCT scan showed the possibility of bilateral lung interstitial inflammation (Figure 2C), and a set of antinuclear antibodies and immune tests showed no abnormality. We continued to give empirical cough relief and expectorant therapy. The third chemotherapy session was postponed until day 31 due to inflammation of the lungs. On the first day of chemotherapy, shortness of breath, dry cough, a small amount of sputum, and difficulty in coughing immediately followed and got progressively worse after the incident. Pulmonary ventilation function was as expected, and severe diffusive dysfunction was found. A joint consultation between the oncology and rheumatology departments diagnosed IIP. After 1 week of hyperbaric oxygen inhalation and anti-bacterial and anti-inflammatory treatments, symptoms did not improve significantly. Treatment with empirical cough and expectorants continued until day 21. On the first day after chemotherapy, the patient experienced shortness of breath, inability to exhale, mild phlegm, pallor, difficulty in coughing, and increased shortness of breath after exercises. Pneumonia was diagnosed at the Department of Respiratory and Critical Care Medicine, and the patient’s condition improved after high-flow oxygen inhalation and antiseptic and anti-inflammatory treatments. One week after chemotherapy, HRCT re-examination revealed bilateral pulmonary interstitial pneumonia, bilateral pleural thickening, and fibrosis (Figure 3), and IPF was diagnosed. After 1 month of anti-infectives and anti-fibrosis treatment with OFEV 150 mg, the pulmonary symptoms were relieved. After a multidisciplinary evaluation, laparoscopic ovarian cancer reduction was performed, and the outcome of the surgery was R1. On the first day after surgery, nab-PTX 400 mg single-agent chemotherapy was administered. Asthma and dyspnea began 36 h after chemotherapy. HRCT examination showed chronic bronchitis with emphysema, interstitial disease of both the lungs, and bronchiectasis of both the lower lobes of the lungs (Figure 4). After continued treatment with methylprednisolone, nintedanib (OFEV), and theophylline sustained-release tablets, the patient recovered and was discharged from the hospital.

## Discussion

Our case is the first to report a rare and life-threatening adverse event in an OC patient. The prognosis for people with ILD is poor. Acute exacerbation (AE) of ILD during chemotherapy can be regarded as a fatal adverse event. There have been reports of related subjects for similar adverse events in a modest number of patients with LC complicated by interstitial pneumonia treated with nab-PTX chemotherapy and in large-scale clinical trials (Otsubo et al., 2017; Fukuizumi et al., 2019). For example, A clinical trial in Europe and the United States showed that 12% of the patients with respiratory system related adverse reactions after the use of albumin paclitaxel chemotherapy in Europe and the United States had dyspnea, 7% had cough, and pneumothorax was rare (<1%). However, only 2% of patients in the Chinese population have dyspnea and cough (Kundranda, and Niu, 2015). Previous studies of continuous paclitaxel safety monitoring have infrequently reported pneumonia, IPF, and pulmonary embolism. In particular, no clinical cases of fatal IPF caused by nab-PTX have been reported in OC patients. However, IPF is a potentially fatal complication of oncology treatment and remains a concern in clinical practice.

In 2018, diagnostic guidelines for IPF were published in the *American Journal of Respiratory Critical Care*. The guidelines state that IPF is a type of chronic progressive fibrotic interstitial pneumonia from an unknown cause. The presence of fibroblasts causes damage to the lung matrix and alveoli, destroying typical lung tissue structures, severely impairing lung function, and even causing death. Treatment is poor and mortality is high, mainly among the elderly; HRCT remains the primary diagnostic clue; and the requirement for lung biopsy is reduced (Zaizen and Fukuoka, 2020). The imaging criteria for ILD are grid-like shadows, irregular linear shadows, and honeycomb shifts around the lower lobes of the lungs and under the pleura. In the classification of unexplained ILD, IPF is the most common type, accounting for more than 60 percent. Chronic inflammation is the fundamental pathological basis (Collins and Luppi, 2021). A detailed medical history is required for patients with unexplained ILD with suspected IPF at the time of initial diagnosis. Diagnosis is recommended by multidisciplinary discussion (MDD) and HRCT. Measurements of serum markers such as MMP-7, sPD, CCL-18, or KL-6 are not recommended to distinguish IPF from other ILDs. At the same time, CTD and alternative ILDs should be significantly excluded from routine serum autoantibodies whenever possible. The chest CT in our case revealed pre-chemo IIP classified as ILD. Studies have shown that ILD is a disease with progressive deterioration of respiratory functions, and previous history of ILD is an independent risk factor for drug-related ILD (Shukuya et al., 2010). After three courses of chemotherapy, our patient’s lung symptoms gradually worsened; IPF diagnosed after MDD is thought to be caused by chemotherapy drugs, with symptoms improving after combined anti-fibrosis therapy.

Nab-PTX is composed of nanoparticles that avoid allergic and other adverse reactions caused by traditional paclitaxel containing toxic solvents such as castor oil (Shitara et al., 2017); (Gradishar et al., 2005). In our case, patients with advanced OC complex IIP had grade II bone marrow depression, fever, fatigue, and additional symptoms 1 week after chemotherapy, which is consistent with reported adverse reactions to nab-PTX (Parisi et al., 2020). The effect of chemotherapy on the patient in our case was remarkable, indicating a significant reduction in tumor size, the disappearance of ascites, and an opportunity for intermediate tumor reduction. However, IIP aggravated during chemotherapy, and IPF was complicated. Patients are treated with nab-PTX monotherapy on the first day after surgery, and AE-ILD emerges. Previous studies have shown that interstitial pneumonia, IPF, and pulmonary embolism have been infrequently reported in the ongoing safety monitoring of paclitaxel. Experimental studies have shown that the simple use of paclitaxel can also lead to lung injury, and relevant animal experiments have also been reported, but the mechanism is still unclear (Pavlovic et al., 2020). Cases of IPF have been rare. After MDD, it was thought to be caused by nab-PTX chemo.

As for the safety of nab-PTX + CBP chemotherapy in the chemotherapy of OC-ILD, there is no relevant report, but there are relevant reports on pulmonary fibrosis during the chemotherapy of LC patients, mainly in NSCLC-ILD and based on radiotherapy and chemotherapy (Fukuizumi, et al., 2019; Liu, et al., 2018). IIP is one of the most common complications in LC patients. The most severe toxicity of chemotherapy is an acute exacerbation of type IIP caused by anti-cancer therapy. Currently, standard chemotherapy is not established for NSCLC-ILD, and nab-PTX + CBP is not included as a treatment contraindication. Some studies have shown that patients with advanced NSCLC-ILD may benefit from nab-PTX + CBP chemotherapy, which is consistent with those without IIP (Yasuda et al., 2018; Fukuizumi et al., 2019; Araya et al., 2019). For NSCLC-ILD patients who cannot undergo surgery, nab-PTX + CBP shows promising efficacy and safety as a first-line chemotherapy regimen and is tolerable in NSCLC patients with mild or moderate ILD (Igawa et al., 2018; Kenmotsu et al., 2019). In addition, OFEV, an intracellular tyrosine kinase inhibitor (TKI) with antibiotic properties, was one of the first drugs approved for use in IPF and has more recently been approved for additional use in chronic fibrosis, ILDs with a progressive phenotype, and systemic sclerosis–associated ILD (SSc-ILD). It is reported that preventive or combined use of OFEV anti-fibrosis therapy can prolong the interval of acute exacerbation of IPF in patients with an elevated risk of tumors in IPF (Fujita et al., 2018; Otsubo et al., 2017). The incidence rate of AE-ILD is acceptable, but the prognosis is limited. Research shows that lung function and AE-ILD may be related to the prognosis of NSCLC-ILD patients (Wang et al., 2020).

ILD is progressive deterioration of respiratory function and an independent risk factor for LC. Epidemiological studies have shown that 22% of people with ILD will eventually develop LC. However, since NSCLC-ILD patients have been excluded from clinical trials, it is uncertain whether chemotherapy benefits these patients. The study reported, platinum drugs are one of the most effective anticancer drugs, and the occurrence of pulmonary fibrosis in patients treated with carboplatin chemotherapy has not been reported before (Schoch et al., 2020). A meta-analysis showed that platinum dual drugs as first-line chemotherapy might be related to the high incidence of AE-ILD (Chen et al., 2015). Some studies have even suggested that patients with advanced NSCLC-ILD detected by chest X-ray examination must be cautiously given nab-PTX + CBP (Shukuya et al., 2010). However, AE-ILD during chemotherapy is generally discussed with drug-specific adverse reactions, and uncontrolled cancer may also be related to the occurrence of AE-ILD (Sekine et al., 2022). In addition, it has been shown that AE-ILD treated with systemic high-dose corticosteroids is significantly associated with in-hospital mortality. Low advanced distributed learning (ADL) score at admission is a risk factor for in-hospital mortality of LC patients receiving systemic chemotherapy (Shiraishi, et al., 2022). Therefore, clinicians should be careful when introducing and selecting chemotherapy regimens for these patients.

The main risk factors of IPF in tumor patients include but are not limited to large tumor load, tumors with high proliferation rate, efficient anti-tumor treatment, liver metastasis, and renal insufficiency. AE-ILD during chemotherapy can be regarded as a fatal adverse event. In the past, no case of fatal IPF in OC patients using nab-PTX has been reported clinically. However, early prevention and identification are essential to reduce mortality because of the seriousness and urgency of pulmonary fibrosis. It is necessary to conduct hierarchical management according to the risk factors and prevent medication to reduce the occurrence of TRAEs. It is worth reminding that once clinicians observe symptoms or auxiliary examination abnormalities related to nab-PTX chemotherapy, multidisciplinary diagnostic evaluation and strict monitoring are required. Accurate and timely diagnosis of IPF and taking appropriate measures helps prevent further deterioration of disease and reduces total mortality. Since nab-PTX was marketed, there have been few cases of IPF, so fatalities are rare. However, attention should be paid to clinical medication. Therefore, for cancer patients with ILD, preventive anti-fibrosis treatment can prolong the time of acute attack and damage to the lungs caused by fibrosis during chemotherapy. Once the diagnosis of IPF is confirmed, anti-tumor treatment or combined anti-pulmonary fibrosis that may lead to IPF should be stopped. Attention should be paid to monitoring the changes in pulmonary function and imaging, properly adjusting the drug dosage and method used, stopping the drug when necessary, and actively treating symptomatically. Patients with ovaries whose genetic testing indicates the presence of BRCA2 mutations typically receive oral short-molecule olaparib at home rather than in the hospital when they receive more effective targeted therapy. Clinicians should also be concerned about emerging adverse events with oral targeted chemotherapy agents, and this is one of the events we will follow in the future.

## Conclusion

In conclusion, our case demonstrates that chemotherapy with nab-PTX in OC-ILD patients can induce and cause IFP. Given the severity of IPF and the widespread use of nab-PTX in the treatment of advanced malignancies, clinicians should maintain a substantial degree of clinical suspicion of ILD. They must first determine the risk level of IPF, assess the toxicity risk of current chemotherapy drugs, and take appropriate steps to minimize irreversible damage from IPF. The pathogenesis of IPF induced by nab-PTX with relation to its safe treatment remains to be further investigated to reduce adverse events and improve treatment safety.

## Data Availability

The original contributions presented in the study are included in the article/Supplementary Material; further inquiries can be directed to the corresponding author.
